# Type 1 diabetes mellitus and non-alcoholic fatty liver disease: a two-sample Mendelian randomization study

**DOI:** 10.3389/fendo.2024.1315046

**Published:** 2024-04-12

**Authors:** Lin Tuo, Li-ting Yan, Yi Liu, Xing-xiang Yang

**Affiliations:** Department of Infectious Disease, Sichuan Provincial People’s Hospital, University of Electronic Science and Technology of China, Chengdu, China

**Keywords:** two-sample Mendelian randomization, type 1 diabetes mellitus, non-alcoholic fatty liver disease, metabolic syndrome, causality

## Abstract

**Background:**

NAFLD (Nonalcoholic fatty liver disease) is becoming an increasingly common cause of chronic liver disease. Metabolic dysfunction, overweight/obesity, and diabetes are thought to be closely associated with increased NAFLD risk. However, few studies have focused on the mechanisms of NAFLD occurrence in T1DM.

**Methods:**

We conducted a two-sample Mendelian randomization (MR) analysis to assess the causal association between T1DM and NAFLD with/without complications, such as coma, renal complications, ketoacidosis, neurological complications, and ophthalmic complications. Multiple Mendelian randomization methods, such as the inverse variance weighted (IVW) method, weighted median method, and MR-Egger test were performed to evaluate the causal association of T1DM and NAFLD using genome-wide association study summary data from different consortia, such as Finngen and UK biobank.

**Results:**

We selected 37 SNPs strongly associated with NAFLD/LFC (at a significance level of *p* < 5 × 10−8) as instrumental variables from the Finnish database based on the T1DM phenotype (8,967 cases and 308,373 controls). We also selected 14/16 SNPs based on with or without complications. The results suggest that the genetic susceptibility of T1DM does not increase the risk of NAFLD (OR=1.005 [0.99, 1.02], IVW p=0.516, MR Egger *p*=0.344, Weighted median *p*=0.959, Weighted mode *p*=0.791), regardless of whether complications are present. A slight causal effect of T1DM without complications on LFC was observed (OR=1.025 [1.00, 1.03], MR Egger *p*=0.045). However, none of the causal relationships were significant in the IVW (*p*=0.317), Weighted median (*p*=0.076), and Weighted mode (*p*=0.163) methods.

**Conclusion:**

Our study did not find conclusive evidence for a causal association between T1DM and NAFLD, although clinical observations indicate increasing abnormal transaminase prevalence and NAFLD progression in T1DM patients.

## 1 Introduction

Non-alcoholic fatty liver disease (NAFLD) has a global prevalence of as high as 25% and poses a significant threat to human health, placing a tremendous economic burden on society (1). However, no pharmacological treatments have been approved for NAFLD worldwide. Metabolic dysfunction, overweight/obesity, and diabetes are thought to be closely associated with increased NAFLD risk. Thus, the latest 2020 international expert consensus statement redefined it as metabolic-associated fatty liver disease (MAFLD) (2, 3). In the progression of type 2 diabetes (T2D) complications, NAFLD can manifest as ectopic triglyceride accumulation through insulin resistance, impaired fatty acid oxidation, and altered lipid metabolism. However, few studies have examined the relationship between T1DM and NAFLD.

T1DM is an autoimmune disease characterized by β-cell destruction and absolute insulin deficiency (4). Treatment of T1DM mainly involves adjusting exogenous insulin supplementation based on the patient’s insulin needs. During T1DM progression, increasing insulin demand and poor glycemic control can lead to indirect insulin resistance (IR) (5). IR induces dyslipidemia through excessive lipolysis, increased lipogenesis, and lipotoxic changes in adipose tissue. Dysregulation of lipid metabolism can cause multi-organ dysfunction including the liver, which is key for NAFLD development. However, the association between T1DM and NAFLD incidence, particularly in advanced T1DM with complications, and its genetic predisposition remains contentious. Conversely, NAFLD is the most common cause of transaminase elevation in T1DM (6, 7), though the underlying mechanisms remain unclear.

Mendelian randomization (MR) analysis uses common genetic variations, typically single nucleotide polymorphisms (SNPs), as instrumental variables (IVs) to explore potential causal relationships between exposures and outcomes (8–10). Since SNPs are randomly assigned at conception and unaffected by confounders (11), they can largely avoid biases from confounding factors. Thanks to rapid GWAS developments and accumulating publicly available GWAS summary statistics, two-sample MR with significantly improved statistical power has become more accessible and flexible. Previous MR studies have elucidated causal relationships between NAFLD, T2D, and obesity (12, 13). However, no MR study has examined the causal association between T1DM and NAFLD.

This article analyzed the causal relationships between T1DM with various complications and NAFLD using a two-sample MR framework, with validation in separate databases.

## 2 Materials and methods

We performed two-sample MR analyses to investigate the causal relationship between type 1 diabetes and non-alcoholic fatty liver disease derived from different large-scale cohorts.

### 2.1 Overview of Mendelian randomization study

**Figure 1:**
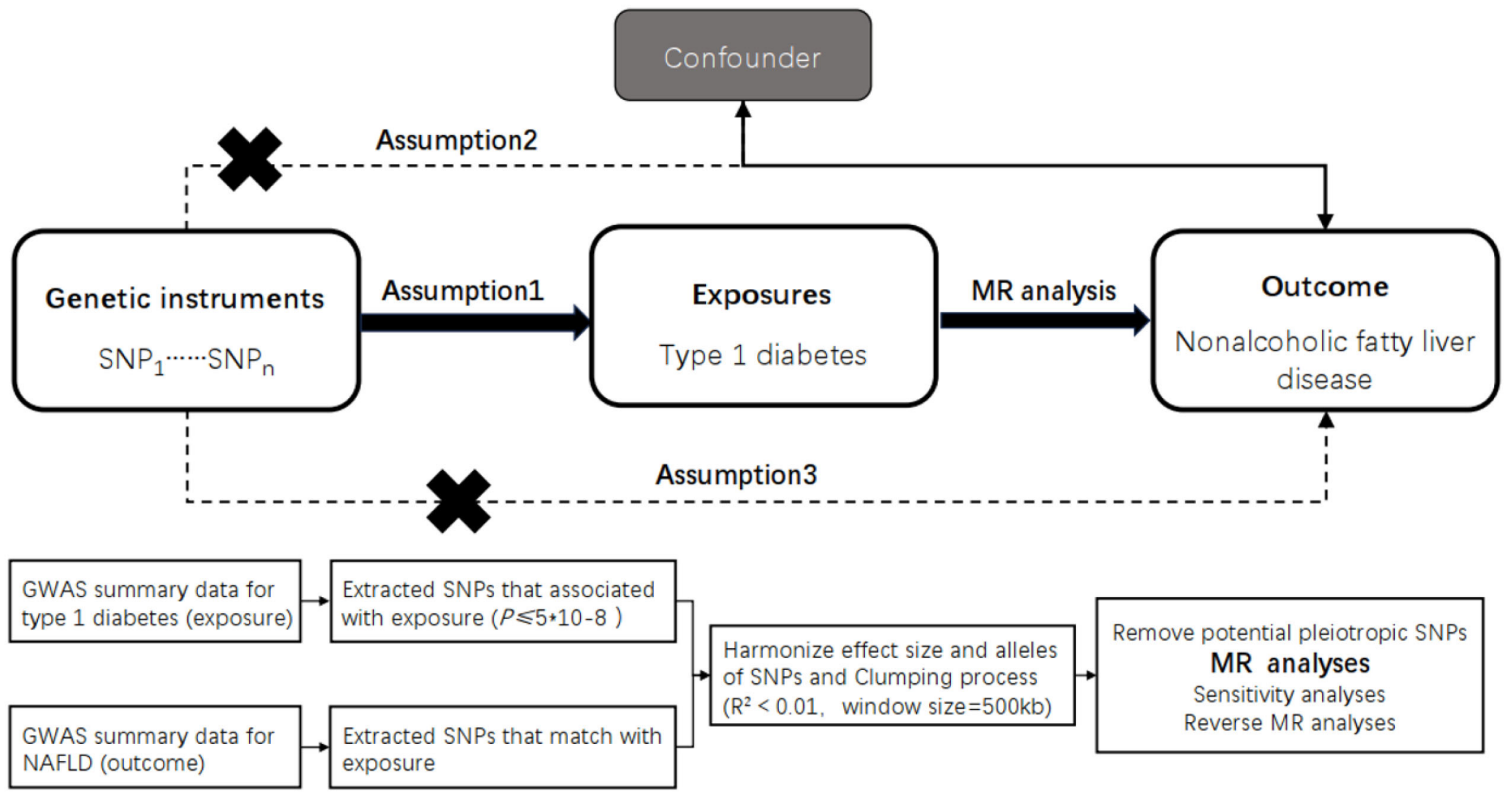
Overview of the design and methods used in this Mendelian randomization study. MR analysis was used to explore the causal relationships, including the following three assumptions: 1. Instrument validity assumption: the genetic variant used as an instrument for the exposure of interest is strongly associated with the exposure but not directly associated with any confounding factors that might influence the outcome. 2. Independence assumption: the genetic variant is independent of any other factors that might influence the outcome, except through its effect on the exposure. 3. Exclusion restriction assumption: the genetic variant affects the outcorne only through its effect on the exposure, and not through any other pathways. The specific workflow employed a Mendelian randomization analysis. The GWAS data for TIDM as the exposure factor and for NAFLD as the outcome factor were collected and inappropriate SNPs were removed through processes such as SNP extraction, harmonization, and clumping, followed by causality analysis. MR, Mendelian randomization; TIDM, type 1 diabetes.

The study design is shown in (**Figure 1**).

We employed the statistical analysis technique known as the two-sample Mendelian randomization (MR) analysis method. This approach utilizes genetic variants as instrumental variables to assess causal relationships between exposure and outcome variables. Mendelian randomization is based on three assumptions: (i) genetic variation is associated with the risk factor, (ii) genetic variation is independent of confounding factors, and (iii) genetic variation influences the outcome only through the risk factor (**Figure 1A**).

The overlap of participants in two-sample Mendelian randomization (MR) may lead to an increased type 1 error rate due to weak instrument bias (14, 15). Non-overlap between exposure and outcome is crucial for the two-sample MR framework. Causal estimates from single-sample analysis (using data from a single data source) tend to be biased toward the observed association between the risk factor and the outcome. In contrast, estimates from two-sample analysis (using data from non-overlapping datasets of the risk factor and the outcome) have a smaller estimation bias, tending towards zero. As most exposure factors were derived from the FinnGen team (https://r9.finngen.fi/), the NAFLD GWAS based on the GWAS catalog (https://www.ebi.ac.uk/) was excluded to avoid bias due to sample overlap.

This analysis was conducted using summary-level data from published genome-wide association studies (GWAS) and followed the STROBE-MR guidelines (9). All GWAS studies included in the analysis had obtained approval from the relevant review committees, and all participants had provided informed consent. The FinnGen biobank GWAS was conducted by the FinnGen team and ethical approval was obtained from the original studies (**Figure 1B**).

### 2.2 Sources of genome-wide association studies

The GWAS summary statistics of NAFLD were obtained from a recently published GWAS catalog (GCST90054782), with 4,761 European NAFLD cases and 373,227 genetically matched controls. Considering NAFLD is closely associated with LFC, a recent LFC GWAS (GCST90016676) which included 25,617 European participants was also selected. The GWAS data for T1DM were obtained from the FinnGen team, which included 8,967 European T1DM cases and 308,373 control cases from the r9 version of the database. Further searches were conducted based on the occurrence of T1DM complications, resulting in GWAS data for the complication group (6,234 cases and 308,280 controls) and the non-complication group (4,918 cases and 183,185 controls). Specific complications were further analyzed, and GWAS data related to coma (2,050 cases), renal dysfunction (4,918 cases), ketoacidosis (2,102 cases), neurological complications (1,077 cases), and optic nerve complications (5,202 cases) were selected (**Table 1**).

**Table 1:** A brief description of GWAS summary statistics. PubMed ID is the ID of publication in the Pubmed.

Overview of databases used for gene-exposure and gene-outcome data					
Class (Consortium)	GWAS dataset	Phenotype	Participants included in analysis	Ethnicity	PubMed ID
gene-exposure (finngen)	T1DM-WIDE	Type 1 diabetes, wide definition	8967 cases and 308373 controls	European ancestry	NA
	E4_DM1NASCOMP	Type 1 diabetes with other specified/multiple/unspecified complications	6234 cases and 308280 controls	European ancestry	NA
	E4_DM1COMA	Type 1 diabetes with coma	2050 cases and 308280 controls	European ancestry	NA
	E4_DM1NOCOMP(R5)	Type 1 diabetes without complications	4918 cases and 183185 controls	European ancestry	NA
	E4_DM1REN	Type 1 diabetes with renal complications	1579 cases and 308280 controls	European ancestry	NA
	E4_DM1KETO	Type 1 diabetes with ketoacidosis	2102 cases and 308280 controls	European ancestry	NA
	E4_DM1NEU	Type 1 diabetes with neurological complications	1077 cases and 308280 controls	European ancestry	NA
	E4_DM1OPTH	Type 1 diabetes with ophthalmic complications	5202 cases and 308280 controls	European ancestry	NA
gene-outcome(GWAS Catalog)	GCST90054782	Nonalcoholic fatty liver disease	4761 cases and 373227 controls	European ancestry	34535985
	GCST90016676	Liver fat content	25617 individuals	European ancestry	34128465

NA is not available.

The coordination of encoding and reference alleles was employed to eliminate ambiguous SNPs with inconsistent alleles in the exposure and outcome datasets. Palindromic SNPs with minor allele frequencies between 0.45 and 0.55 were defined as having ambiguous minor alleles and all potentially ambiguous SNPs were excluded in sensitivity analyses. Some missing tools were not imputed by proxy SNPs, as the impact of a small fraction of missing tools on the results is considered negligible.

### 2.3 Statistical analysis

Mendelian randomization (MR) assumes that the genetic variants used as instruments are closely related to the exposure of interest and are unrelated to any potential confounding factors that might influence the outcome. The *F*-statistic quantifies the strength of association of each assumed risk factor with its genetic instrument. The power of each SNP was evaluated by its *F*-statistic (*F* = beta2/se2) and the overall *F*-statistic for all SNPs was calculated (excluding SNPs with *F*-values less than 10) (**Table 2**). Statistical power was calculated using mRnd (https://shiny.cnsgenomics.com/mRnd/). Additionally, the PhenoScanner (V2) database was utilized for filtering genetic variants associated with confounding factors. Subsequently, these rigorously filtered SNPs were employed as final instrumental variables for subsequent Mendelian randomization analysis. In this study, no instrumental variables related to phenotypes such as insulin resistance and lipid metabolism alterations were identified.

**Table 2:** *F* statistic and power estimation.

*F* statistic and power estimation				
Class	Phenotype	SNPs	R2(%)	*F* statistic
Exposure	T1DM-WIDE	37	1.85%	161.89
	E4_DM1NASCOMP	14	7.03%	2584.66
	E4_DM1COMA	8	15.46%	16961.62
	E4_DM1NOCOMP(R5)	16	5.74%	921.73
	E4_DM1REN	6	16.90%	27791.79
	E4 DM1KETO	8	16.97%	33078.65
	E4 DM1NEU	4	28.84%	51632.29
	E4 DM1OPTH	15	8.41%	3193.69

T1DM GWAS published in GWAS Catalog before June 1, 2023. R2 is the variance of phenotype explained by SNPs; SNPs are the number of single nucleotide polymorphisms.

We conducted and reported the results of a range of sensitivity analyses that allowed for effective estimation in the presence of horizontal pleiotropy. First, the random-effects inverse variance weighted method was used to relax the exclusion restriction assumption by allowing all SNPs to exhibit random pleiotropic effect while maintaining asymptotic unbiasedness even if all SNPs show horizontal pleiotropy (16). The random-effects inverse variance weighted method has been reported as the most common and best-performing method in various scenarios that violate the exclusion restriction principle (17). Second, if at least 50% of the selected SNPs are valid, the weighted median estimator can provide an unbiased causal effect (18). Third, the MR-PRESSO method was performed, which can detect pleiotropy outliers in the multiple instrument summary-level MR analysis, and obtain causal effect estimates using the inverse variance weighted method after excluding the outliers (19). Finally, leave-one-out sensitivity tests were conducted to examine the influence of peripheral and pleiotropic SNPs on causal estimates (20). Heterogeneity and pleiotropy of individual SNPs were assessed using Cochran’s Q statistic and MR-PRESSO. Finally, a fixed-effects model was used to combine the MR results from the discovery and replication stages. All statistical analyses were performed using R 4.1.3 (R Foundation for Statistical Computing, Vienna, Austria).

## 3 Results

### 3.1 SNP selection

Firstly, several SNPs were identified as IVs (instrumental variables) related to different phenotypes in various databases, with a strong correlation (at a significance level of *p* < 5 × 10−8). Among the wide-definition T1DM phenotypes, 37 SNPs were identified. Based on the T1DM with/without complications, 14/16 SNPs were selected. Additionally, 5-15 SNPs were identified as related phenotypes of different T1DM complications. Secondly, the variances of all the aforementioned SNPs ranged from 1.85% to 28.84%. Eventually, the *F* statistics of all IVs were greater than 10, indicating the absence of potential weak instrument bias (**Table 2**).

### 3.2 The causal association between T1DM with/without complications and NAFLD

**Figure 2:**
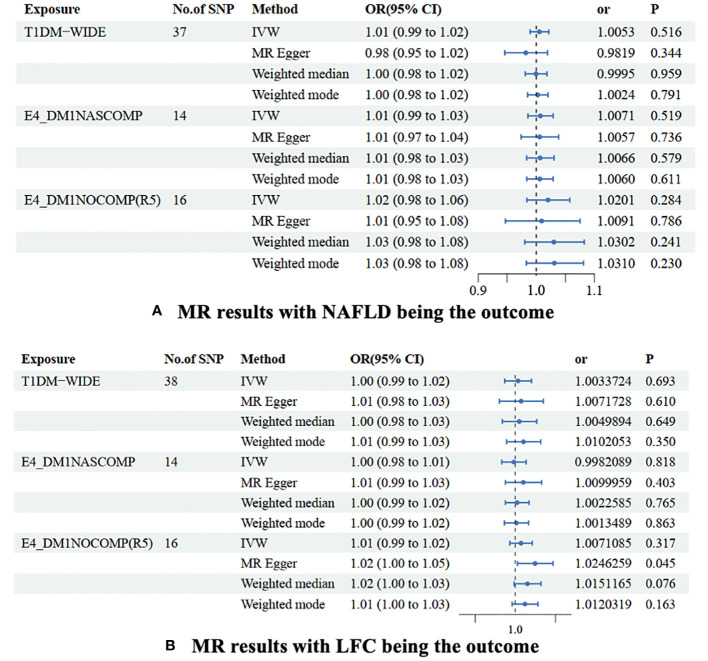
MR results were non-alcoholic fatty liver disease **(A)** and liver fat content **(B)** as the outcomes. The association of TIDM and NAFLD/LFH was analyzed with four different MR methods. NAFLD is a non-alcoholic fatty liver disease; LFC is liver fat content. No. of SNP is the number of single nucleotide polymorphisms used in MR analysis; OR is the odds ratio; 95% CI is the limit of 95% confidence interval of OR/BETA; SE is standard error; P is the *p*-value of OR. TIDM-WIDE is type 1 diabetes, wide definition; E4_DMINASCOMP is type 1 diabetes with other specified/multiple/unspecified complications; E4_DMINOCOMP is type 1 diabetes without complications.

Generally, our MR study suggested that there might be no causal relationship between NAFLD and T1DM. Preliminary findings indicate that the genetic susceptibility of T1DM does not increase the risk of NAFLD (OR=1.005 [0.99, 1.02], IVW *p*=0.516). Consistent conclusions were obtained through additional statistical methods such as MR Egger (OR=0.982 [0.95, 1.02], *p*=0.344), Weighted median (OR=1.000 [0.98, 1.02], *p*=0.959), and Weighted mode (OR=1.002 [0.98, 1.02], *p*=0.791). Considering that T1DM is often classified into the presence or absence of complications, subgroup analysis for these two phenotypes also suggests that the genetic predisposition of both phenotypes does not influence the risk of NAFLD, both for T1DM with complications (OR=1.007 [0.99, 1.03], IVW *p*=0.519) and without complications (OR=1.020 [0.98, 1.06], IVW *p*=0.284) (**Figure 2A**, **Supplementary Table 1**).

A slight causal effect of T1DM without complications on LFC was observed (OR=1.025 [1.00, 1.03], MR Egger *p*=0.045). However, none of the causal relationships were significant in the IVW (*p*=0.317), Weighted median (*p*=0.076), and Weighted mode (*p*=0.163) methods. Similarly, genetically-driven T1DM (OR=1.003 [0.99, 1.02], IVW *p*=0.693) and T1DM with complications (OR=0.998 [0.98, 1.01], IVW *p*=0.818) did not alter LFC. In the MR-Egger regression and weighted median methods, none of the causal relationships were significant (**Table 3**). Despite detecting slight heterogeneity in the T1DM-NAFLD and T1DM-LFC analyses, the conclusions remained valid after outlier removal (**Figure 2B**, **Supplementary Table 2**).

**Table 3:** MR results of MR-Egger regression and weighted-median method.

MR results of MR-Egger regression and weighted-median method												
Exposure	Outcome	NSNP	MR-Egger				Weighted-Median				Pheterogeneity	Ppleiotropy
			OR(BETA)	95%LCI	95%UCI	P	OR(BETA)	95%LCI	95%UCI	P		
T1DM-WIDE	NAFLD	37	0.981912	0.94596	1.019228	0.344043	0.9995066	0.98104	1.018323	0.95863351	0.4922661	0.1795833
E4_DM1NASCOM P	NAFLD	14	1.0056694	0.97395	1.038423	0.735532	1.0066417	0.98338	1.030456	0.57895236	0.6031378	0.9066822
E4_DM1COMA	NAFLD	8	1.009528	0.9652	1.055892	0.693322	1.0092368	0.98473	1.034352	0.4634836	0.3802523	0.9979506
E4_DM1NOCOMP (R5)	NAFLD	16	1.0090524	0.94675	1.07546	0.785737	1.0301534	0.98024	1.082613	0.24108568	0.673851	0.6897835
E4_DM1REN	NAFLD	5	1.0209431	0.98482	1.058392	0.341492	1.0073415	0.98302	1.03226	0.55739514	0.569773	0.2893432
E4_DM1KETO	NAFLD	8	1.0120135	0.97729	1.047967	0.527503	1.0099209	0.98618	1.034238	0.41608169	0.8533245	0.9996891
E4_DM1NEU	NAFLD	4	0.960329	0.89382	1.03179	0.384166	1.0062078	0.97822	1.034992	0.66715852	0.4222276	0.2949585
E4_DM1OPTH	NAFLD	15	1.0268997	0.98194	1.073913	0.266039	1.0080403	0.97653	1.040564	0.62110279	0.2121181	0.1916191
T1DM-WIDE	LFC	38	1.0071728	0.98014	1.034949	0.609751	1.0049894	0.9837	1.026738	0.64865631	0.1737316	0.7302244
E4_DM1NASCOM P	LFC	14	1.0099959	0.98752	1.032981	0.40329	1.0022585	0.98753	1.017209	0.76523273	0.1030269	0.2001517
E4_DM1COMA	LFC	8	0.9970144	0.97764	1.016777	0.775334	1.000683	0.98791	1.013619	0.91702069	0.7881714	0.4914296
E4_DM1NOCOMP (R5)	LFC	16	1.0246259	1.00266	1.047075	0.045096	1.0151165	0.99844	1.03207	0.07583278	0.2376233	0.0742224
E4_DM1REN	LFC	6	1.0024586	0.98017	1.025254	0.840974	1.0005349	0.98745	1.013794	0.93655074	0.3271541	0.9748787
E4_DM1KETO	LFC	8	1.0031379	0.98605	1.020525	0.733065	1.0009862	0.98893	1.01319	0.87333089	0.6235146	0.9521878
E4_DM1NEU	LFC	4	0.991399	0.96034	1.023463	0.647961	1.0015483	0.98954	1.013701	0.80148595	0.511194	0.5297885
E4_DM1OPTH	LFC	14	1.0075646	0.97914	1.036815	0.614414	1.0037588	0.98704	1.020765	0.6616132	0.1147365	0.74051

NSNP is the number of single nucleotide polymorphisms; OR, odds ratio; 95%LCI, the lower limit of 95% confidence interval; 95%UCI, the upper limit of 95% confidence interval; P is the p-value of OR(BETA); Pheterogeneity is the p-value of heterogeneity test from Cochrane’s Q value; Pleiotropy is the p-value of pleiotropy test from MR-Egger intercept.

### 3.3 The causal association between common T1DM with different complications and NAFLD

#### 3.3.1 E4_DM1COMA is type 1 diabetes with coma

**Figure 3:**
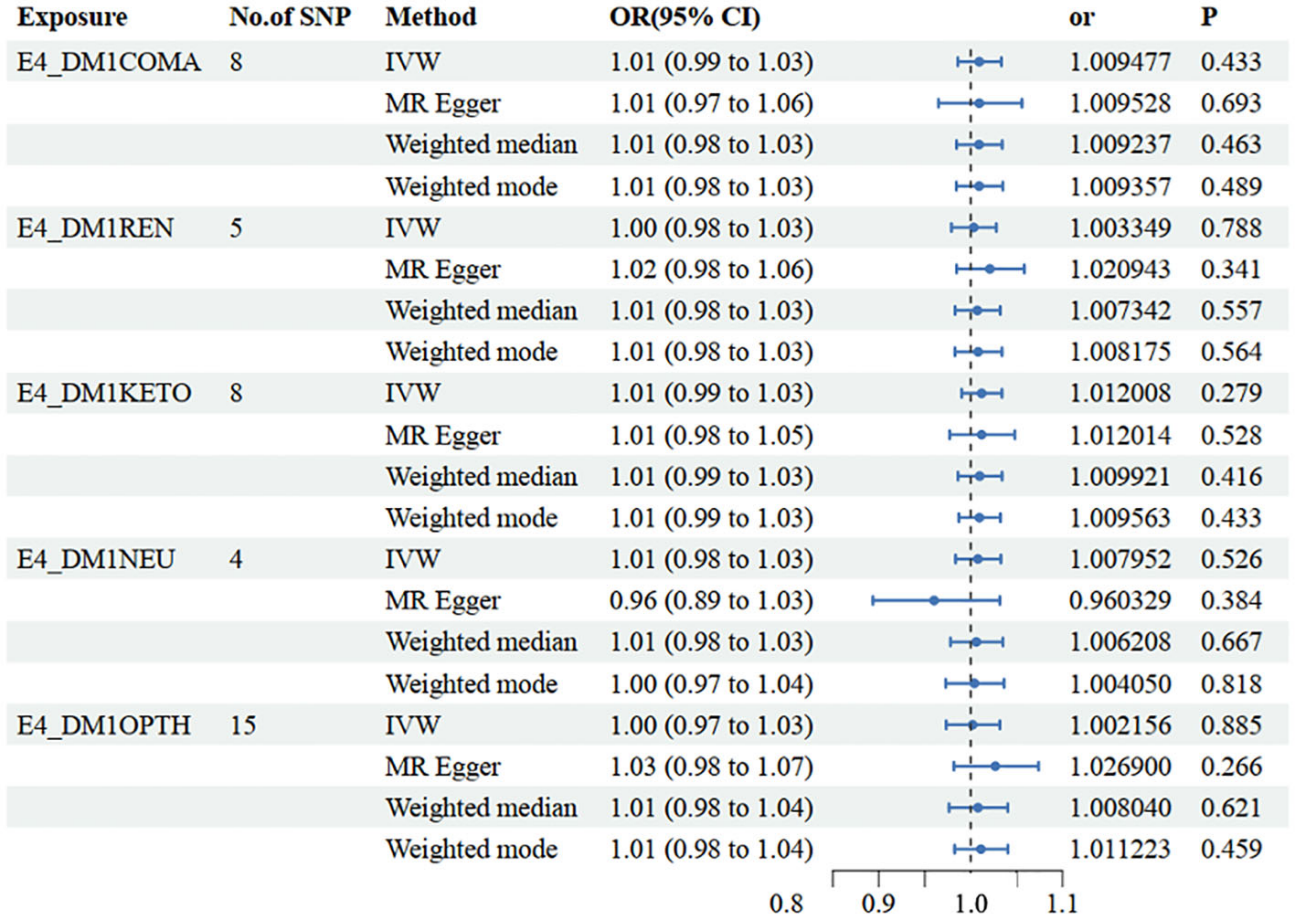
Mendelian randomization results of causal effects between type 1 diabetes with different complications and NAFLD. E4_DMICOMA is type 1 diabetes with coma; E4_DMIREN is type 1 diabetes with renal complications; E4_DMIKETO is type 1 diabetes with ketoacidosis; E4_DMINEU is type 1 diabetes with neurological complications; E4_DM1OPTH is type 1 diabetes with ophthalmic complications.

In the set of IVs (*p* < 5 × 10−8), 8 SNPs related to type 1 diabetes with coma were identified. It was found that no genetic liability to type 1 diabetes with coma was causally associated with NAFLD (OR=1.009 [0.99, 1.03], IVW *p*=0.433). Consistent conclusions were obtained through additional statistical methods such as MR Egger (OR=1.010 [0.97, 1.06], *p*=0.693), Weighted median (OR=1.010 [0.98, 1.03], *p*=0.463), and Weighted mode (OR=1.009 [0.98, 1.03], *p*=0.489) (**Figure 3**, **Supplementary Table 1**).

**Figure 4:**
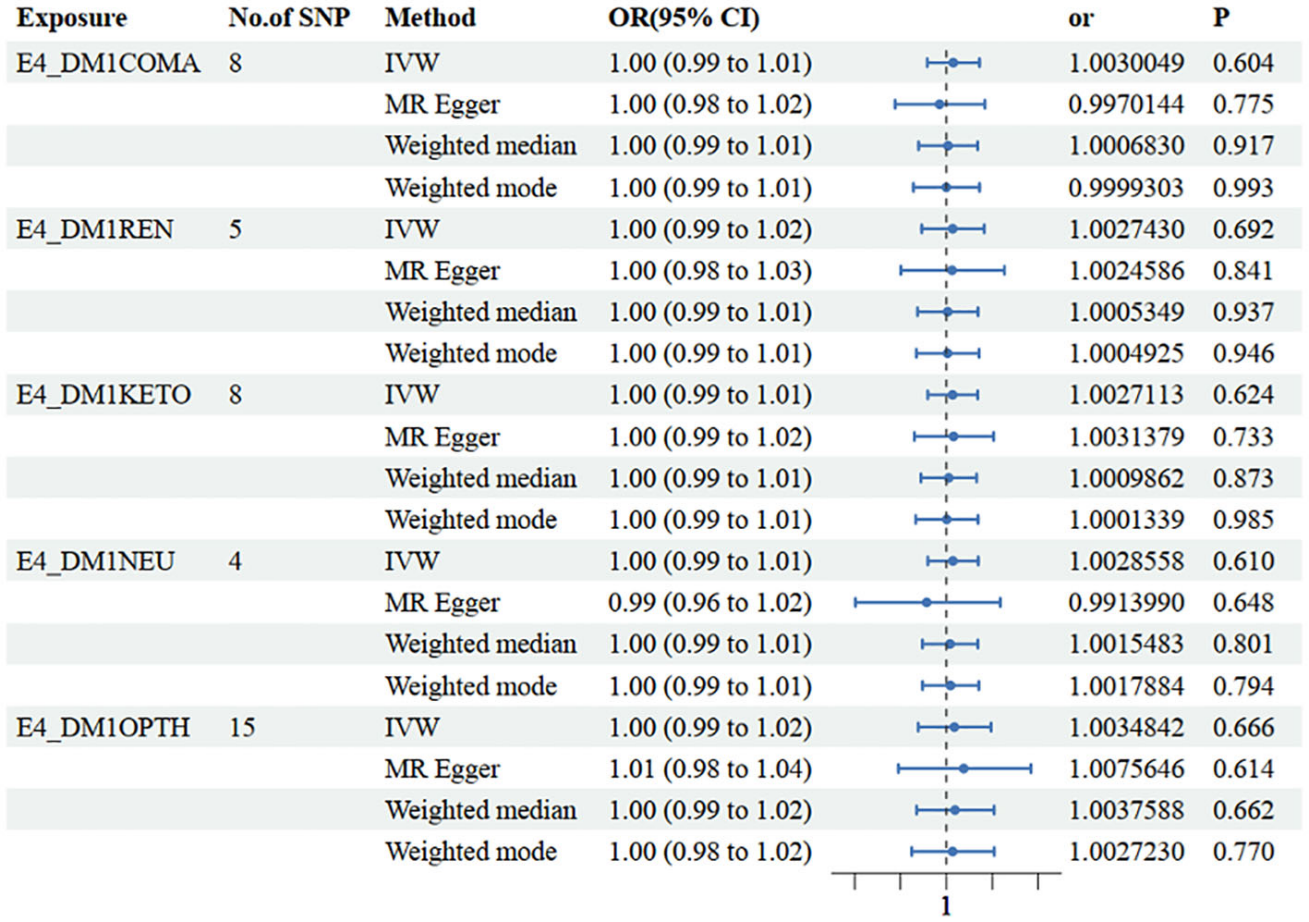
Mendelian randomization results of causal effects between type 1 diabetes with different complications and LFH.

In the other database, consistent conclusions were obtained between type 1 diabetes with coma and LFC (OR=1.003 [0.99, 1.01], IVW *p*=0.604) (**Figure 4**, **Supplementary Table 2**).

#### 3.3.2 E4_DM1REN is type 1 diabetes with renal complications

In the set of IVs (*p* < 5 × 10−8), 5 SNPs related to type 1 diabetes with renal complications were identified. It was found that no genetic liability to type 1 diabetes with renal complications was causally associated with NAFLD (OR=1.003 [0.98, 1.03], IVW *p*=0.788). Consistent conclusions were obtained through additional statistical methods such as MR Egger (OR=1.021 [0.98, 1.06], *p*=0.341), Weighted median (OR=1.007 [0.98, 1.03], *p*=0.557), and Weighted mode (OR=1.008 [0.98, 1.03], *p*=0.564) (**Figure 3**, **Supplementary Table 1**).

In the other database, consistent conclusions were obtained between type 1 diabetes with renal complications and LFC (OR=1.003 [0.99, 1.02], IVW *p*=0.692) (**Figure 4**, **Supplementary Table 2**).

#### 3.3.3 E4_DM1KETO is type 1 diabetes with ketoacidosis

In the set of IVs (*p* < 5 × 10−8), 8 SNPs related to type 1 diabetes with ketoacidosis were identified. It was found that no genetic liability to type 1 diabetes with ketoacidosis was causally associated with NAFLD (OR=1.012 [0.99, 1.03], IVW *p*=0.279). Consistent conclusions were obtained through additional statistical methods such as MR Egger (OR=1.012 [0.98, 1.05], *p*=0.528), Weighted median (OR=1.010 [0.99 1.03], *p*=0.416), and Weighted mode (OR=1.010 [0.99, 1.03], *p*=0.433) (**Figure 3**, **Supplementary Table 1**).

In the other database, consistent conclusions were obtained between type 1 diabetes with ketoacidosis and LFC (OR=1.003 [0.99, 1.01], IVW *p*=0.624) (**Figure 4**, **Supplementary Table 2**).

#### 3.3.4 E4_DM1NEU is type 1 diabetes with neurological complications

In the set of IVs (*p* < 5 × 10−8), 4 SNPs related to type 1 diabetes with neurological complications were identified. It was found that no genetic liability to type 1 diabetes with neurological complications was causally associated with NAFLD (OR=1.008 [0.98, 1.03], IVW *p*=0.526). Consistent conclusions were obtained through additional statistical methods such as MR Egger (OR=0.960 [0.89, 1.03], *p*=0.384), Weighted median (OR=1.010 [0.98 1.03], *p*=0.667), and Weighted mode (OR=1.004 [0.97, 1.04], *p*=0.818) (**Figure 3**, **Supplementary Table 1**).

In the other database, consistent conclusions were obtained between type 1 diabetes with neurological complications and LFC (OR=1.003 [0.99, 1.01], IVW *p*=0.610) (**Figure 4**, **Supplementary Table 2**).

#### 3.3.5 E4_DM1OPTH is type 1 diabetes with ophthalmic complications

In the set of IVs (*p* < 5 × 10−8), 15 SNPs related to type 1 diabetes with ophthalmic complications were identified. It was found that no genetic liability to type 1 diabetes with ophthalmic complications was causally associated with NAFLD (OR=1.002[0.97, 1.03], IVW *p*=0.885). Consistent conclusions were obtained through additional statistical methods such as MR Egger (OR=1.027 [0.98, 1.07], *p*=0.266), Weighted median (OR=1.008 [0.98 1.04], *p*=0.621), and Weighted mode (OR=1.011 [0.98, 1.04], *p*=0.459) (**Figure 3**, **Supplementary Table 1**).

In the other database, consistent conclusions were obtained between type 1 diabetes with ophthalmic complications and LFC (OR=1.004 [0.99, 1.02], IVW *p*=0.666) (**Figure 4**, **Supplementary Table 2**).

## 4 Discussion

Given the escalating global burden of metabolic disorders, conducting high-quality prospective research to evaluate the correlation between the genetic condition T1DM and metabolic disorder NAFLD presents significant challenges. Our study extensively examined the correlation between non-alcoholic fatty liver disease (NAFLD) and type 1 diabetes mellitus (T1DM). Through an in-depth analysis of a substantial and independent cohort, our findings suggest that there may not be a direct causal relationship between T1DM and NAFLD. Prior studies have posited a plausible link between NAFLD and T1DM, potentially attributed to metabolic disruptions stemming from the evolution of T1DM, including secondary metabolic derangements induced by T1DM complications (21). We further investigated the relationship between NAFLD and T1DM with complications. Our research found that the presence or absence of complications such as renal dysfunction, coma, ketoacidosis, neurological complications, and retinopathy did not increase the probability of NAFLD development in individuals with T1DM. In other words, regardless of complications, the genetic susceptibility of T1DM does not increase the likelihood of NAFLD. Our findings carry noteworthy implications for understanding the T1DM-NAFLD relationship, potentially facilitating the clinical management of T1DM patients with concurrent NAFLD.

The observed causal effect of T1DM on LFC in this study may be a false positive, as this result was not significant in both the IVW and weighted median methods. However, we cannot completely rule out this causal relationship, as the genetic susceptibility of T1DM, high glucose-induced oxidative stress, and cellular metabolic dysfunction may impact the pathogenesis of NAFLD (22, 23). Additionally, reports suggest that as the disease progresses, the degree of hepatic steatosis increases further in T1DM with complications (21, 24, 25). Therefore, further investigations are warranted to elucidate this association.

Regarding the null associations, several factors can explain them. Metabolic syndrome, characterized by obesity, hyperglycemia, dyslipidemia, and systemic hypertension, currently represents the most prominent risk factor for NAFLD (26). More than 70% of NAFLD patients typically manifest elevated levels of triglycerides (TG), total cholesterol (TC), low-density lipoprotein cholesterol (LDL-C), and reduced levels of high-density lipoprotein cholesterol (HDL-C), primarily synthesized in the liver, indicating dysregulated lipid metabolism in these individuals. In contrast to type 2 diabetes mellitus (T2DM), well-controlled patients with type 1 diabetes mellitus (T1DM) show no significant alterations in lipid levels (LDL-C, HDL-C, TG) and blood pressure levels (27–29). Furthermore, the majority of T1DM patients tend to develop a lean phenotype rather than obesity (29). Hence, we postulate that T1DM may induce a specific metabolic state that may not predispose individuals to NAFLD.

Interestingly, there is direct evidence suggesting that the absolute insulin deficiency in T1DM patients, leading to the absence of portal vein/peripheral insulin gradients, may reduce insulin stimulation of hepatic lipogenesis and impede the development of NAFLD (30). Additionally, *Perseghin et al.* (31) reported significantly lower hepatic fat content in T1DM patients compared to non-diabetic individuals, with no significant association between NAFLD and T1DM (32). However, due to the lifelong subcutaneous insulin injections required in T1DM, relative peripheral hyperinsulinemia and hepatic hypoinsulinemia may occur, leading to insulin resistance (33). This relative insulin-resistant state can affect glucose and lipid hepatic metabolism, triggering pro-inflammatory cascades and resulting in hepatic steatosis, fibrosis, and cirrhosis (34–36). The onset and progression of NAFLD can be promoted following insulin resistance in T1DM. Therefore, the observed coexistence of NAFLD and T1DM may be attributed to the effects of treatment, particularly the impact of insulin resistance on NAFLD. In conclusion, based on previous studies, it is challenging to demonstrate a direct promotion of NAFLD development by T1DM.

Furthermore, recent studies indicate an association between NAFLD and complications in patients with T1DM (21). In the general population, the prevalence of hepatic steatosis (defined as HSI > 36) is 37.1%, but it increases to 49% in T1DM patients with complications and to 32% in those without complications. This suggests an independent correlation of NAFLD with cardiovascular disease, chronic kidney disease, retinopathy, and neuropathy in T1DM patients, regardless of established risk factors (24, 25, 37, 38). Some researchers propose that poor blood glucose control in patients with chronic diabetes complications leads to the conversion of glucose into fat in the liver via GLUT2 transport, thereby promoting hepatic lipid accumulation (39). However, these studies are based on populations of poorly controlled T1DM patients with complications, and there is no difference in prevalence compared to normal overweight populations. From a genetic susceptibility perspective, it cannot be inferred that the occurrence of T1DM (including T1DM with complications) is causally related to the development of NAFLD.

The selection of instrumental variables is a critical step in Mendelian randomization studies. To ensure validity, any genetic variation must influence the outcome solely through its effect on the exposure, without horizontal pleiotropy (40, 41). To address potential pleiotropic factors, MR-Egger regression can be used to reveal a statistically significant intercept when these genetic variants are excluded, thereby reducing heterogeneity and eliminating horizontal pleiotropy (42). In patients with T1DM, metabolic disturbances resulting from insulin resistance and alterations in glucose and lipid metabolism may significantly contribute to the development of NAFLD (43, 44). In this study, according to the fundamental assumption of MR studies, we adopted SNPs with a significance threshold of P < 5 × 10^-8 from the GWAS data as instrumental variables. To address potential linkage disequilibrium effects on the analysis outcomes, we imposed criteria of r^2 < 0.001 and a window size of 10,000 kb. To ensure robust associations between instrumental variables and endogenous variables and to mitigate bias from weak instrumental variables, we computed R^2 [R^2 = 2 × EAF × (1 - EAF) × b^2], which represents the proportion of variance explained by instrumental variable SNPs, and the F-statistic [F = R^2 × (N - 2)/(1 - R^2)] to evaluate the strength of instrumental variables, each with its distinct characteristics. Furthermore, we utilized the PhenoScanner (V2; http://www.phenoscanner.medschl.cam.ac.uk/upload/) database to filter out genetic variants linked to potential confounding factors like insulin resistance. These meticulously screened SNPs were subsequently employed as the definitive instrumental variables for subsequent MR analyses.

To minimize potential weak instrument bias amplification, separate databases with minimal sample overlap between exposure and outcome data sources were utilized. Despite our efforts, limitations of this study have been acknowledged that must be taken into account. One limitation is the challenges of pleiotropy in the MR framework, as found in all MR studies (45, 46). To address this, various sensitivity analyses with different assumptions were conducted.

Our research boasts several noteworthy strengths. Firstly, this analysis enrolled the largest number of phenotypes of T1DM with complications in different datasets to date, including discovery and validation stages, thereby enhancing the persuasiveness of the findings (47). Secondly, we conducted rigorous sensitivity analyses to validate the IVs pertinent to our hypothesis. After considering horizontal pleiotropy, outliers, and sample overlap, the confidence of the results was increased. Thirdly, our study was restricted to individuals of European descent, which could reduce population stratification bias (48, 49).. Finally, in our study, we maintained a minimal degree of overlap between exposure and outcome data sources, so that inflation of the weak instrument bias could be avoided as much as possible.

## 5 Conclusion

In this study, a two-sample Mendelian randomization framework was utilized to investigate the potential causal relationship between T1DM and NAFLD. The analysis was validated subsequent to the integration and scrutiny of selected databases. Our findings suggest that T1DM, as well as combined with complications, does not directly lead to an increased incidence of NAFLD. Mechanisms linking the two beyond genetics remain unclear and warrant further investigation in future studies.

## Data availability statement

The original contributions presented in the study are included in the article/**Supplementary Material**. Further inquiries can be directed to the corresponding authors.

## Ethics statement

The GWAS used in the current study were approved by their relevant review board, and written informed consent for participation was not required for the work in accordance with the national legislation and institutional requirements.

## Author contributions

LT: Data curation, Writing – original draft. LY: Formal analysis, Funding acquisition, Writing – review & editing. YL: Writing – review & editing. XY: Formal analysis, Writing – review & editing.
