# Supplementary material for: Type 1 diabetes mellitus and non-alcoholic fatty liver disease: a two-sample Mendelian randomization study
Source: Front Endocrinol (Lausanne). 2024 Apr 12;15:1315046. doi: 10.3389/fendo.2024.1315046 (PMC11045944; doi:10.3389/fendo.2024.1315046)
Supplement: Supplementary file 1 [file DataSheet_1.pdf]

Table S1. The analysis result of TIDM and NAFLD was analyzed with four different MR methods.

Exposure	Outcome	SNPs	IVW				MR-Egger				Weighted median				Weighted mode			
			OR(beta)	95%LCI	95%UCI	*P*	OR(beta)	95%LCI	95%UCI	*P*	OR(beta)	95%LCI	95%UCI	*P*	OR(beta)	95%LCI	95%UCI	*P*
T1DM-WIDE	NAFLD	37	1.0053	0.9894	1.0215	0.5161	0.9819	0.9460	1.0192	0.3440	0.9995	0.9810	1.0183	0.9586	1.0024	0.9850	1.0201	0.7910
E4_DM1NASCOMP	NAFLD	14	1.0071	0.9856	1.0291	0.5186	1.0057	0.9739	1.0384	0.7355	1.0066	0.9834	1.0305	0.5790	1.0060	0.9836	1.0289	0.6108
E4_DM1COMA	NAFLD	8	1.0095	0.9860	1.0335	0.4327	1.0095	0.9652	1.0559	0.6933	1.0092	0.9847	1.0344	0.4635	1.0094	0.9844	1.0349	0.4892
E4_DM1NOCOMP(R5)	NAFLD	16	1.0201	0.9837	1.0579	0.2837	1.0091	0.9467	1.0755	0.7857	1.0302	0.9802	1.0826	0.2411	1.0310	0.9829	1.0813	0.2297
E4_DM1REN	NAFLD	5	1.0033	0.9792	1.0281	0.7879	1.0209	0.9848	1.0584	0.3415	1.0073	0.9830	1.0323	0.5574	1.0082	0.9829	1.0341	0.5641
E4_DM1KETO	NAFLD	8	1.0120	0.9904	1.0341	0.2791	1.0120	0.9773	1.0480	0.5275	1.0099	0.9862	1.0342	0.4161	1.0096	0.9872	1.0325	0.4333
E4_DM1NEU	NAFLD	4	1.0080	0.9836	1.0329	0.5259	0.9603	0.8938	1.0318	0.3842	1.0062	0.9782	1.0350	0.6672	1.0041	0.9728	1.0363	0.8184
E4_DM1OPTH	NAFLD	15	1.0022	0.9733	1.0318	0.8849	1.0269	0.9819	1.0739	0.2660	1.0080	0.9765	1.0406	0.6211	1.0112	0.9826	1.0407	0.4595

IVW is inverse-variance weighted; SNPs is the number of single nucleotide polymorphisms; OR is odds ratio; 95%LCI is the lower limit of 95% confidence interval; 95%UCI is the upper limit of 95% confidence interval; P is the p-value of OR.
