# Supplementary material for: Type 1 diabetes mellitus and non-alcoholic fatty liver disease: a two-sample Mendelian randomization study
Source: Front Endocrinol (Lausanne). 2024 Apr 12;15:1315046. doi: 10.3389/fendo.2024.1315046 (PMC11045944; doi:10.3389/fendo.2024.1315046)
Supplement: Supplementary file 2 [file DataSheet_2.pdf]

Table S2. The analysis result of TIDM and LFH was analyzed with four different MR methods.

Exposure	Outcome	SNPs	IVW				MR-Egger				Weighted median				Weighted mode			
			OR(beta)	95%LCI	95%UCI	*P*	OR(beta)	95%LCI	95%UCI	*P*	OR(beta)	95%LCI	95%UCI	*P*	OR(beta)	95%LCI	95%UCI	*P*
T1DM-WIDE	NAFLD	38	1.00337244	0.98676053	1.02026401	0.69264528	1.00717278	0.98014226	1.03494876	0.60975098	1.00498941	0.98370151	1.02673799	0.64865631	1.01020526	0.98919779	1.03165887	0.34977843
E4_DM1NASCOMP	NAFLD	14	0.99820888	0.983107	1.01354274	0.81770994	1.00999589	0.98752257	1.03298065	0.40329036	1.00225849	0.98752726	1.01720947	0.76523273	1.00134894	0.9864076	1.0165166	0.86319989
E4_DM1COMA	NAFLD	8	1.0030049	0.99170402	1.01443457	0.60376075	0.99701441	0.97763564	1.01677731	0.77533391	1.00068297	0.98791252	1.0136185	0.91702069	0.99993026	0.9856051	1.01446363	0.99270597
E4_DM1NOCOMP(R5)	NAFLD	16	1.00710845	0.99323746	1.02117317	0.31680612	1.02462586	1.00265798	1.04707504	0.04509561	1.01511654	0.99844116	1.03207043	0.07583278	1.01203189	0.99600035	1.02832148	0.16274141
E4_DM1REN	NAFLD	6	1.00274299	0.98922229	1.01644848	0.69248443	1.00245864	0.98016979	1.02525434	0.8409736	1.00053485	0.98744889	1.01379423	0.93655074	1.00049247	0.98691843	1.01425321	0.94642048
E4_DM1KETO	NAFLD	8	1.00271132	0.99190443	1.01363596	0.62431207	1.0031379	0.98604723	1.0205248	0.73306506	1.00098618	0.98892945	1.0131899	0.87333089	1.00013387	0.98663665	1.01381573	0.98513257
E4_DM1NEU	NAFLD	4	1.00285583	0.99193716	1.01389469	0.60964548	0.99139899	0.96033918	1.02346334	0.64796133	1.0015483	0.98954161	1.01370068	0.80148595	1.00178841	0.98959053	1.01413664	0.79358033
E4_DM1OPTH	NAFLD	15	1.00348418	0.98775916	1.01945954	0.66602211	1.00756459	0.97913967	1.0368147	0.6144143	1.00375884	0.9870357	1.02076532	0.6616132	1.002723	0.98492778	1.02083973	0.77034036
