# Supplementary material for: Type 1 diabetes mellitus and non-alcoholic fatty liver disease: a two-sample Mendelian randomization study
Source: Front Endocrinol (Lausanne). 2024 Apr 12;15:1315046. doi: 10.3389/fendo.2024.1315046 (PMC11045944; doi:10.3389/fendo.2024.1315046)
Supplement: Supplementary file 3 [file DataSheet_3.pdf]

PTP Table

PTP Table. Detailed information of instrumental variables utilized in the MR analysis of the causal effects of T1DM on NAFLD.

SNP	Effect allele	Other allele	Se	Beta	P	F
rs115048884	T	C	0.0405787	0.676378	2.23E-62	277.8322699
rs115204205	C	T	0.0419177	0.264625	2.74E-10	39.85353523
rs115221241	A	G	0.065514	-0.492361	5.68E-14	56.48056824
rs12928537	A	G	0.0172469	-0.11948	4.28E-12	47.99194346
rs147392974	A	G	0.0517609	-0.435449	4.01E-17	70.77354618
rs151233	T	C	0.0229046	0.136321	2.65E-09	35.42257292
rs1591805	G	A	0.0153436	0.0890258	6.55E-09	33.66489263
rs181316459	C	G	0.0400223	-0.22258	2.68E-08	30.92916462
rs1872424	C	T	0.0157945	-0.0906896	9.37E-09	32.9688052
rs1990760	T	C	0.0158947	0.106056	2.52E-11	44.5210928
rs2289702	T	C	0.0239844	-0.131116	4.58E-08	29.88502794
rs2476601	G	A	0.0196745	-0.44637	5.93E-114	514.7336832
rs2516390	T	C	0.0166407	0.468946	1.01E-174	794.1499067
rs281379	A	G	0.0157047	0.105862	1.58E-11	45.4381771
rs28449420	C	T	0.0605302	-0.791967	4.07E-39	171.1866738
rs2847273	C	A	0.0157564	-0.10445	3.38E-11	43.94433468
rs28896571	T	C	0.0387698	-0.5274	3.82E-42	185.0517215
rs3087243	A	G	0.0167364	-0.168952	5.82E-24	101.906663
rs3095250	T	C	0.0150898	0.460346	1.00E-200	930.6829985
rs3184504	C	T	0.0156567	-0.151947	2.87E-22	94.18544622
rs34337125	A	G	0.0159171	-0.123605	8.13E-15	60.30373177
rs35914000	A	G	0.0158683	0.0871909	3.91E-08	30.19127932
rs35947132	A	G	0.0457191	-0.268058	4.54E-09	34.37654301

rs402072	C	T	0.023045	-0.12671	3.83E-08	30.23210289
rs4711369	A	G	0.0156074	-0.166373	1.57E-26	113.6330264
rs4755391	T	G	0.0181189	-0.10193	1.85E-08	31.64757168
rs60888743	G	A	0.0187815	-0.104384	2.73E-08	30.88924696
rs689	T	A	0.0206414	0.460279	3.79E-110	497.2376691
rs6908626	T	G	0.0229956	0.180912	3.63E-15	61.89353081
rs7034200	A	C	0.0155537	0.0901234	6.86E-09	33.57435662
rs7090530	A	C	0.0168467	0.134216	1.63E-15	63.47151998
rs7130222	G	T	0.0180637	-0.100921	2.31E-08	31.21401728
rs74203920	T	C	0.0383473	0.314781	2.24E-16	67.38254954
rs76288224	G	A	0.0209817	-0.424462	5.32E-91	409.2571597
rs7823699	G	A	0.0163794	-0.0950068	6.62E-09	33.64444768
rs876498	A	G	0.0162334	0.117002	5.70E-13	51.94785157
rs9391997	G	A	0.015299	0.103025	1.65E-11	45.3475136

MR, Mendelian Randomization; T1DM, Type 1 diabetes; SNP, single nucleotide polymorphism; SE, standard error.
